# Esketamine in Treatment Resistant Depression: Acute Effects on Dissociation and Therapeutic Effects at the End of the Induction Period

**DOI:** 10.1192/j.eurpsy.2025.867

**Published:** 2025-08-26

**Authors:** G. Lombardozzi, G. D. Kotzalidis, G. Trovini, G. Albanesi, G. Civita, D. Donato, V. Giovanetti, S. De Filippis

**Affiliations:** 1 Psychiatry, Villa von Siebenthal Neuropsychiatric Clinic, Genzano di Roma, Roma, Italy

## Abstract

**Introduction:**

Esketamine has been linked to dissociation, which was claimed to predict or not to predict antidepressant response. Speculations regarding predictivity were based on results obtained with the CADSS, a scale investigating dissociative symptoms, with higher scores indicating more symptoms.

**Objectives:**

To investigate the effect of intranasal esketamine on dissociation and its subsequent influence on clinical response, we administered the CADSS 40 minutes after inhalation at the first esketamine administration and 40 minutes after the ninth inhalation and measured clinical response through the Clinical Global Impressions-Severity (CGI-S), the Young Mania Rating Scale (YMRS), the Montgomery-Åsberg Depression Rating Scale (MADRS), and the 24-item Brief Psychiatric Rating Scale (BPRS).

**Methods:**

We included 61 adults (33 women and 28 men; age, mean, 52.69±11.80, range 20-73 years) with Thase & Rush (J Clin Psychiatry 1997;58 [Suppl 13]:23-29) treatment-resistant depression. All patients received intranasal esketamine spray and were assessed at the first and ninth administrations (one month after the first administration, i.e., the end of the induction period and the beginning of maintenance), at the day of the spray after 40 min with the CADSS and 1 month later with the CGI-S, the BPRS, the MADRS, and the YMRS.

**Results:**

CADSS scores dropped from 6.59±7.42 40 min after the first administration to 3.12±4.41 40 minutes 1 month later (dropped by 3.48 points, 47.27% of baseline); *t*=3.15; *p*=0.0021. CGI-S scores dropped from 5.12±0.61 at baseline to 3.95±0.76 1 month later (*t*=9.32; *p*<0.00001). BPRS scores dropped from 51.85±11.55 at baseline to 41.21±10.64 after 1 month (*t*=5.29; *p*<0.00001). MADRS scores dropped from 34.29±7.89 at baseline to 22.61±9.07 after 1 month (*t*=7.59; *p*<0.00001). Responders (≥50% drop of MADRS from baseline) were 12 patients (19.67%), while remitters (MADRS score≤10) were 2 (3.28%). YMRS scores moved from 2.18±2.59 at baseline to 1.72±2.37 1 month later (*t*=1.02; *p*=0.309, n.s.), always in the normal range. Blood pressure 40 minutes after spray at the first administration was unchanged in 35 patients, increased in 16 (maximum by 20 mmHg), and decreased in 10 (maximum drop 20 mmHg). Contrary to previous claims, CADSS scores did not correlate at any time with scores on clinical scales or therapeutic response (Pearson’s *r* from 0.232 with *p*=0.072 to 0.013 with *p*=0.918). As for side effects, 15 patients reported dissociation, 15 sedation, 8 vertigo, 8 dizziness, 6 confusion, 5 headache, 4 nausea/vomiting, and 0 hypertension.

**Conclusions:**

Patients in our sample scored very low on the CADSS. At the end of the induction period, esketamine was associated with significant decreases in the severity of psychopathology.

**Disclosure of Interest:**

None Declared

